# Supplementary material for: A dataset of human and robot approach behaviors into small free-standing conversational groups
Source: PLoS One. 2021 Feb 25;16(2):e0247364. doi: 10.1371/journal.pone.0247364 (PMC7906375; doi:10.1371/journal.pone.0247364)
Supplement: S1 Table — (PDF) [file pone.0247364.s001.pdf]

S1 Table. Overview of the CongreG8 dataset.

Dataset Name	CongreG8
Origin	Stockholm, Sweden
License	Creative Commons Attribution-NonCommercial-ShareAlike 4.0
Download URL	https://sites.google.com/view/congreg8
Research description	Understanding and modelling group behaviours within and newcomer behaviours to free-standing conversational groups
Data collection method	Motion capture recording sessions; game scenario; WoZ robot; questionnaires
Trial length	2-6 seconds
Condition 1	
Name	Human-group condition
Description	Human newcomer approaches group of three humans
Participants	40 (27F:13M); aged 22 to 35 (M=25.8, SD=3.2)
Trials	380 trials
Condition 2	
Name	Robot-group condition
Description	Robot newcomer (1 Pepper robot) approaches group of three humans
Participants	16 from the main group of 40 participants
Trials	38 trials
Data Component 1	
Description	Questionnaires for personality (BFI-10), Godspeed (GQS), human-robot and human-human questionnaires
Data type	Likert scale
File type	XLSX (Microsoft Excel Open XML Spreadsheet)
Number of files	1
Size (uncompressed)	15 kb
Data Component 2	
Description	Processed motion capture data
Data type	3D corrected marker positions and 3D position and rotation (in quaternion format) of reconstructed human skeletons or robot rigid body
File type	CSV (comma-separated values)
Number of files	424
Size (uncompressed)	4.95 GB
Data Component 3	
Description	Processed motion capture data
Data type	3D corrected marker positions and 3D position and rotation reconstructed human skeletons or robot rigid body
File type	FBX (Autodesk Filmbox)
Number of files	412
Size (uncompressed)	1.05 GB
Data Component 4	
Description	Raw motion capture data

Data type	37 markers per group member/human newcomer recorded @ 120 fps.
File type	TAK (OptiTrack Motive) + calibration files
Number of files	380
Size (uncompressed)	754 GB
