# Supplementary material for: A dataset of human and robot approach behaviors into small free-standing conversational groups
Source: PLoS One. 2021 Feb 25;16(2):e0247364. doi: 10.1371/journal.pone.0247364 (PMC7906375; doi:10.1371/journal.pone.0247364)
Supplement: S2 Table — Rows labelled “Average 1”-“Average 10” represent average values for each pool of four participants respectively. (PDF) [file pone.0247364.s002.pdf]

S2 Table. The big-five personality traits (E, A, C, N, O) and group behavior labels (Accommodate percentage A% and Ignore percentage I%) across all groups. Rows labelled “Average 1”-“Average 10” represent average values for each *pool* of four participants respectively.

ID	Member	E	A	C	N	O	Label (A%/I%)
1	Group	4.17	4.00	4.33	3.83	4.83	12.50/87.50
	Newcomer	4.00	5.00	3.00	4.00	4.00	
2	Group	4.67	4.33	4.00	4.33	4.50	33.33/66.67
	Newcomer	2.50	4.00	4.00	2.50	5.00	
3	Group	4.17	3.83	4.00	3.17	4.67	33.33/66.67
	Newcomer	4.00	5.50	4.00	6.00	4.50	
4	Group	3.50	4.83	3.67	4.17	4.50	0.00/100.00
	Newcomer	6.00	2.50	5.00	3.00	5.00	
Average 1	4 participants	4.13	4.25	4.00	3.88	4.63	19.45/80.55
5	Group	4.67	5.50	4.33	5.33	4.17	0.00/100.00
	Newcomer	5.00	6.00	4.00	2.50	6.00	
6	Group	5.33	5.67	4.33	4.33	4.83	0.00/100.00
	Newcomer	3.00	5.50	4.00	5.50	4.00	
7	Group	4.50	5.67	4.17	4.33	4.67	0.00/100.00
	Newcomer	5.50	5.50	4.50	5.50	4.50	
8	Group	4.50	5.67	4.17	4.50	4.53	9.09/90.91
	Newcomer	5.50	5.50	4.50	5.00	4.00	
Average 2	4 participants	4.75	5.63	4.25	4.63	4.63	2.43/97.57
9	Group	4.33	5.83	5.17	4.83	4.00	10.00/90.00
	Newcomer	3.00	6.00	5.50	5.00	6.00	
10	Group	4.50	5.83	5.00	4.50	4.50	30.00/70.00
	Newcomer	2.50	6.00	6.00	6.00	4.50	
11	Group	3.50	5.83	5.67	5.50	4.83	10.00/90.00
	Newcomer	5.50	6.00	4.00	3.00	3.50	
12	Group	3.67	6.00	5.17	4.67	4.67	33.33/66.67
	Newcomer	5.00	5.50	5.50	5.50	4.00	
Average 3	4 participants	4.00	5.88	5.25	4.88	4.50	21.43/78.57
13	Group	4.33	5.50	5.33	5.00	5.50	0.00/100.00
	Newcomer	5.50	5.00	6.00	3.50	6.50	
14	Group	4.67	5.17	5.50	4.67	6.17	33.33/66.67
	Newcomer	4.50	6.00	5.50	4.50	4.50	
15	Group	4.83	5.67	5.00	4.17	5.50	30.00/70.00
	Newcomer	4.00	4.50	7.00	6.00	6.50	
16	Group	4.67	5.17	6.17	4.67	5.83	36.36/63.64
	Newcomer	4.50	6.00	3.50	4.50	5.50	
Average 4	4 participants	4.63	5.38	5.50	4.63	5.75	25.00/75.00
17	Group	3.17	6.67	4.83	4.83	5.00	70.00/30.00
	Newcomer	3.50	4.50	4.00	4.50	5.50	
18	Group	2.50	6.00	3.83	4.17	5.00	66.67/33.33
	Newcomer	5.50	6.50	7.00	6.50	5.50	
19	Group	3.33	6.00	4.83	4.50	4.83	80.00/20.00
	Newcomer	3.00	6.50	4.00	5.50	6.00	
20	Group	4.00	5.83	5.00	5.50	5.67	91.67/8.33

Continued on next page

Table 2 – continued from previous page

ID	Member	E	A	C	N	O	Label (A%/I%)
	Newcomer	1.00	7.00	3.50	2.50	3.50	
Average 5	4 participants	3.25	6.13	4.63	4.75	5.13	78.05/21.95
21	Group	4.33	5.00	4.33	3.83	6.00	20.00/80.00
	Newcomer	5.50	4.00	7.00	7.00	7.00	
22	Group	4.00	5.00	5.17	5.17	6.83	20.00/80.00
	Newcomer	6.50	4.00	4.50	3.00	4.50	
23	Group	5.83	4.67	5.17	5.50	6.17	40.00/60.00
	Newcomer	1.00	5.00	4.50	2.00	6.50	
24	Group	4.33	4.33	5.33	4.00	6.00	18.18/81.82
	Newcomer	5.50	6.00	4.00	6.50	7.00	
Average 6	4 participants	4.63	4.75	5.00	4.63	6.25	24.39/75.61
25	Group	4.67	4.33	5.83	3.83	4.83	20.00/80.00
	Newcomer	6.50	7.00	4.50	3.00	4.50	
26	Group	5.83	5.33	5.33	4.00	4.67	50.00/50.00
	Newcomer	3.00	4.00	6.00	2.50	5.00	
27	Group	5.00	5.33	5.50	2.83	4.50	77.78/22.22
	Newcomer	5.50	4.00	5.50	6.00	5.50	
28	Group	5.00	5.00	5.33	3.83	5.00	41.67/58.33
	Newcomer	5.50	5.00	6.00	3.00	4.00	
Average 7	4 participants	5.13	5.00	5.50	3.63	4.75	46.34/53.66
29	Group	4.33	5.67	5.00	4.17	4.67	40.00/60.00
	Newcomer	2.50	4.50	5.00	4.00	4.50	
30	Group	3.67	5.67	5.50	4.33	4.67	30.00/70.00
	Newcomer	4.50	4.50	3.50	3.50	4.50	
31	Group	3.33	5.33	4.83	3.50	4.50	66.67/33.33
	Newcomer	5.50	5.50	5.50	6.00	5.00	
32	Group	4.17	4.83	4.67	4.50	4.67	50.00/50.00
	Newcomer	3.00	7.00	6.00	3.00	4.50	
Average 8	4 participants	3.88	5.38	5.00	4.13	4.63	46.34/53.66
33	Group	3.67	4.67	4.67	3.50	4.33	50.00/50.00
	Newcomer	2.50	5.00	5.50	5.50	6.00	
34	Group	2.50	4.67	4.50	4.50	4.67	100.00/0.00
	Newcomer	6.00	5.00	6.00	2.50	5.00	
35	Group	4.00	4.83	5.67	4.17	5.17	90.00/10.00
	Newcomer	1.50	4.50	2.50	3.50	3.50	
36	Group	3.33	4.83	4.67	3.83	4.83	90.91/9.09
	Newcomer	3.50	4.50	5.50	4.50	4.50	
Average 9	4 participants	3.38	4.75	4.88	4.00	4.75	82.93/17.07
37	Group	4.83	5.83	4.50	5.00	6.33	40.00/60.00
	Newcomer	3.00	5.00	4.50	3.00	6.00	
38	Group	4.33	5.33	4.50	4.33	6.00	100.00/0.00
	Newcomer	4.50	6.50	4.50	5.00	7.00	
39	Group	4.33	6.00	4.50	4.83	6.67	0.00/100.00
	Newcomer	4.50	4.50	4.50	3.50	5.00	
40	Group	5.00	4.83	5.17	5.17	5.83	25.00/75.00
	Newcomer	5.50	6.50	4.50	6.50	7.00	
Average 10	4 participants	4.38	5.63	4.50	4.50	6.25	35.48/64.52
