# Supplementary material for: A dataset of human and robot approach behaviors into small free-standing conversational groups
Source: PLoS One. 2021 Feb 25;16(2):e0247364. doi: 10.1371/journal.pone.0247364 (PMC7906375; doi:10.1371/journal.pone.0247364)
Supplement: Attachment — Submitted filename: Response to Reviewers.pdf [file pone.0247364.s003.pdf]

RESPONSE TO REVIEWERS

In the following, we address the reviewers' comments in the same order as in the review.

Comment:

1. Please ensure that your manuscript meets PLOS ONE's style requirements, including those for file naming. The PLOS ONE style templates can be found at

2. We note that Figures [1,4,5] includes an image of a [patient / participant / in the study].

Response: We have checked the paper and filenames to ensure that they comply with PLOS ONE's style and naming requirements. We also obtained appropriate PLOS ONE specific consent for individuals appearing in the images in the paper (Figures 1,4,5) and added the following text in the paper to clarify this:

"The individuals depicted in the images in this manuscript have provided their informed consent (as outlined in PLOS consent form) to publish these case details."

Reviewer #1:

Comment*:* "Although it is original, the dataset has several limitations: The dataset focuses on a fixed group configuration (4 participants only) and a specific interaction scenario/a role-play setting. It is not clear how algorithms trained such a controlled and limited dataset can be scaled to practical applications with varying conversational group configurations, especially for the use cases discussed in the paper."

Response: From our observation of available datasets containing free-standing conversation groups ([7-9]), groups with three and four members tend to be the most common in the types of situation that our dataset is intended to be applied to (small, free-standing conversational groups). While it is indeed limited to conversational group activities and may not scale to large groups or groups involved in different task types, e.g. oriented towards a poster on a wall, the core aim was to take what could be considered a standard, but limited situation into consideration in a detailed and accurate manner with respect to motion data capture. We do intend to increase the dataset in

the future to include other group types and activities that may not share the same characteristics as those in the current dataset. This also includes additional HRI data collection activities in which the robot trajectories will be data-driven based on information from this dataset. The application of the dataset, and its scope, have been demonstrated in the use cases in references [39] and [52].

Comment: “The number of participants/groups is limited (40 participants/10 groups only). Approaching behavior has been captured for the same group several times, which introduces its own biases as after a couple interactions the participants will already become acquainted.”

Response: We randomly assigned participants to each participant pool (i.e. group of four participants with revolving roles between game payer and adjudicator) to attempt to reduce the effects of acquaintance existing prior to the study. However, it is correct that in any interaction like this, participants may become acquainted over the course of the interaction. To check for this, we have included an analysis (see S2 Table and Fig. 14) and text that suggests Accommodate behaviors did not increase as the experiment progressed.

Comment: “It is not clear from the paper what is aimed to be captured. If they aim to capture the tendency of a group of people to accept a newcomer, is the designed experiment appropriate for this aim?”

The aim has been to collect an accurate and detailed set of behaviors related to small group interactions, especially data related to positioning, orienting and full-body motions as a newcomer approaches and joins the group. In the coffee break or poster session scenarios captured in previous datasets [7-9], for example, the joining group behaviors are very rare, even over long collection periods. Our scenario attempts to model a practical, real-world situation, in the sense that group members are engaged in a conversation and do not know when and where the newcomer will approach to join the group, while at the same time enabling as many samples as possible of joining behaviors to be collected. We have clarified this aim in the abstract of the paper and the motivation for the scenario design in the last paragraph of the “Data collection scenario” section.

Comment: "The paper does not provide further insight into why humans prefer a human newcomer over a robot newcomer. This might be due to the robot's limited capabilities for maintaining interaction during the game. The description of the robot control is very brief in the paper, and it is not clear what behaviour is automatic/what behaviour is controlled by the human operator."

Response: According to some participants, they were not familiar with robots and were afraid the robot might collide with them. A WoZ approach was chosen since there is no good automatic control system for moving a robot into an appropriate position in a dynamic group situation. In WoZ, the robot's position is controlled by the teleoperator, and it results in non-smooth trajectories. CongreG8's main purpose is to provide data of human group approach interactions as a basis for training machine learning models for robots approaching groups of humans in a socially compliant manner and then to replace the WoZ control. We have extended the description of robot control in the "Robot control" section of the paper.

Comment: "The dataset mainly focuses on motion capture data. What are practicalities of motion capture data in real-life scenarios, where it is not possible to ask humans to wear mobcap suits? Also, how can this approach be implemented on a robot to perceive and approach a group?"

Response: Compared with extracting skeleton data from videos, we choose full-body motion capture as it offers many accurate data with less occlusion and higher continuity. The high-quality CongreG8 dataset is useful in training data-driven models. Once the model is trained, it could be applied to video inputs, and skeleton extraction methods could be used to extract markers from videos before inputting to the model. CongreG8 additionally offers video streams if researchers prefer to train models using videos.

When it comes to how it could be implemented in a robot to perceive the group, we have demonstrated an approach in [52] in which the full-body marker data (from surrounding cameras) are input into the trained model in real-time. In the future, group information could potentially be extracted from the robot's camera in real-time, although there are several additional challenges associated with doing that.

Comment: “The authors should include a section summarizing the data statistics. It would be helpful for the reader to see some visualizations of how group use the space, how much they move, how they move when a newcomer joins the group, etc. Further statistics regarding the distribution of labels in particular, personality, accommodate and ignore should be presented in the paper.”

Response: We have included a new table (S1 Table) in the supporting documentation that summarizes the statistic of the overall dataset. Statistics regarding the distribution of labels and visualizations of groups are added in the paper, including a new figure (Figure 10) with a heatmap to visualize the positions of all group members in the across all trials in the dataset, in addition to sample images corresponding full body behaviors and newcomer trajectories. As mentioned in a previous response, we also added a new table (S2 Table) in the supporting documentation to summarize the distribution of personality and group behavior labels, in addition to a new figure (Figure 14) to show the relationship between Accommodation labels and personality.

Comment: “The authors should discuss the following datasets/papers in the related work section:

- JRDB: A Dataset and Benchmark for Visual Perception for Navigation in Human Environments
- Robot-centric perception of human groups
- RICA: Robocentric Indoor Crowd Analysis Dataset
- Robocentric Conversational Group Discovery”

Response: Thank you for the references. They have been added to the paper in addition to some explanatory text in the “Group interaction datasets” section of the paper.

Reviewer #2:

Comment: “The related work lists the relevant literature.

There are a few papers that the authors could add to the "Group interaction research" section if they believe it adds to their paper: …”

Rebuttal: Thank you for the suggested references. We have included them in the "Group interaction research" section.

Comment: "In the context of publishing the dataset as a scientific paper, I would encourage the authors to add further analysis of the collected data, for example as described in line 400: the CongreG8 dataset could be analyzed further to discover the relation between the self-reported personality and actually performed behaviors in groups."

Rebuttal: We have added further analysis, including text, data visualizations and statistics in the paper (S1 Table and S2 Table, Figures 10 and 14) in order to better explain the dataset. We also added a discussion of limitations that includes the personality and behaviors in groups**.**

Comments: "Grammatical errors:

(Please note that I am not a English native speaker.)

189 - In the Greetings stage ...

192 - In the meanwhile, a/the face tracker is ...

397 - It helps [to] generate better robot behaviors and [to] minimize the difference between robot-group and human-group interactions."

Rebuttal: Thank you for highlighting these errors. We have corrected them and also had a native English speaker proof-read numerous important parts of the paper to make improvements to writing quality and clarity.
